# A volunteer-led approach to physical activity at large scale—the example of the Gaelic Athletic Organisation

**DOI:** 10.1093/eurpub/ckad214

**Published:** 2024-04-03

**Authors:** Anthony Staines, Emer O’Hara

**Affiliations:** School of Nursing, Psychotherapy and Community Care, Dublin City University, Dublin 9, Ireland; Faculty of Science and Health, Dublin City University, Dublin 9, Ireland

There is substantial evidence that physical activity at any age is beneficial to human health. A wide variety of health states are improved by increasing cardiovascular fitness, strength and/or flexibility. Exercise programs and policies, local, regional, national and international, have been devised for every age, for men and women, and for people with disabilities.^1^ Despite this inactivity remains common, and participation in regular physical activity is relatively low.^2^

There are many possible responses to these challenges, but we describe a response which has been sustained for over 120 years, based entirely on the voluntary activity of thousands of people across one small country. Three associations cover this. The Gaelic Athletic Association, the GAA, (1884), the Camogie Association (1904) and the Ladies Gaelic Football Association (1974). It was with respect and appreciation of this movement that we hosted the social activity of the 2023 EPH conference in Croke Park, the GAA headquarters and main stadium. Table 1 gives the approximate sizes of each organization.

**Table 1:** The approximate sizes of each organization

Code	Clubs	Membership	Active players
Gaelic Athletic Association	2000 (500 overseas)	650 000	200 000
Camogie Association	600	100 000	Not available
Ladies Gaelic Football Association	1000	200 000	Not available
Total	3600	950 000	–

These associations work together, and there is a proposal for a merger. They manage three main sports codes, hurling and Gaelic football for men, camogie for women, and Gaelic football for women. They are based in clubs at the parish level which mostly compete within their own county. Within clubs, players are streamed by age from under 10s to seniors. At the county level, there is a county team, and counties compete with neighbouring counties, both nationally and internationally.

While there are differences in structure between the three sports bodies, the general outline is the same. Clubs were, and are, set up by local volunteers, supported by the county, and with some support at the national level. Each club secures its own playing space, develops the necessary facilities and buildings and trains and supports a range of teams, from young children to adults. It is common for the three codes to be part of one club and share one set of facilities. These sports are not confined to the island of Ireland, and there are many active clubs across the world. Most were established by Irish emigrants, although they attract a much wider range of participants, but a number are wholly indigenous to their communities.

The impact of these sports bodies has been studied occasionally, although there seem to be no published systematic analyses. Some examples of public health-related work on Gaelic games include an effective physical activity motivation programme for younger girls^3^; and the effects of community support on women volunteer coaches.^4^ There is one very large study of coaches, which found that coaches spent almost 10 h a week on club-related activity, a large majority had a coaching qualification, and that about one in five were women.^5^ These are all clear benefits.

What is perhaps less clear is the impact on population activity. Very large numbers of young people engage in Gaelic field sports, but it is not clear that a high proportion of adults do so.^6^ Overall participation in sports and exercise is lower in women, and in those from lower socio-economic groups.^6^ These are not dissimilar to the figures from Eurostat.^2^ Most of the many thousands of coaches are highly committed and intend to continue to coach.^5^

While there are significant initiatives to reach out to older potential players, the limited evidence suggests that the main impact of Gaelic games is on younger people, and coaches. Perhaps the two most striking features of these bodies are their strong local identity, and the high level of commitment of the typical volunteer. These games and their structures provide an affordable and sustainable model for the promotion of physical activity in the population, are being developed to encompass a wider range of participants and should be further studied.

*Conflicts of interest*: None declared.
